# Commencements

**Published:** 1896-08

**Authors:** 


### COMMENCEMENTS.

### Detroit College of Medicine.

The third annual commencement exercises of the Department of Dental Surgery of the Detroit College of Medicine were held at the Lyceum Theatre, Thursday evening, June 4, 1896.

The proceedings were opened with a selection from "Martha" by the orchestra, after which Rev. S. W. Horner engaged in prayer.

Interesting addresses were delivered by Professor J. Taft, of the University of Michigan, and Professor H. O. Walker, of the Detroit College of Medicine.

Hon. Sidney D. Miller conferred the degrees.

A gold medal, the gift of Dr. E. C. Skinner, was presented by that gentleman to Dr. G. H. Lau, of the graduating class.

Dr. W. G. Merdian read the class prophecy, and Dr. G. H. Lau delivered the valedictory.

At intervals, throughout the evening, several vocal and instrumental selections were rendered.

The following is a list of the graduates :

| NAME. | RESIDENCE. | NAME. | RESIDENCE. |
|---|---|---|---|
| F E Bush | Missouri | A B McEachren | Ontario |
| A W Evans | Ontario | F R Nice | Michigan |
| R J Farmer | Michigan | C O'Keefe | Michigan |
| A Foster | Ontario | J Pulford | Ontario |
| H M Lamb | Michigan | G P Ritchie | Michigan |
| S H Large | Ontario | J E Roche | Michigan |
| G H Lau | Pennsylvania | H L Scott, M.D | Michigan |
| W G Merdian | Michigan | F J Sheldon | Michigan |
| H Milloy | Ontario | A W Stewart | Ontario |

### Richmond University College of Medicine.

Commencement exercises were held April 29, 1896, in the Academy of Music, Richmond, Virginia.

The address to the graduating class was delivered by ex-Governor Fitzhugh Lee, Consul-General to Havana, Cuba.

Diplomas were presented by Hunter McGuire, M.D., LL.D., President.

Prizes were delivered by Stuart McGuire, M.D., Professor of Principles of Surgery.

The total number of dental students in attendance during the year was thirty-six.

The following is a list of the graduates :

| NAME. | RESIDENCE. | NAME. | RESIDENCE. |
|---|---|---|---|
| F V Clarke | Virginia | J W Marshall | Virginia |
| W J Cowardin | Virginia | E C McSparran | Virginia |
| George D Farrow | Virginia | Charles A Newland | Virginia |
| Frank Ferguson | South Carolina | A Cary Oppenheimer | Virginia |
| M T Gay | Virginia | William Pilcher | Virginia |
| John H Hartman, jr. | Virginia | E U Potter, jr | Virginia |
| W R Lynn | Iowa | | |

### Western Reserve University—Dental Department.

The annual commencement exercises of the Dental Department of Western Reserve University was held May 19, 1896, at Association Hall, Cleveland, Ohio, at 7:30 P. M.

Rev. J. M. Buckley, of New York City, delivered the address.

The degree was conferred by President Charles F. Thwing.

The Adelbert Glee Club rendered the music for the occasion.

A banquet followed the exercises.

The number of students during the session was fifty-three.

The following is a list of the graduates.

| NAME. | RESIDENCE. |
|---|---|
| William George Ebersole | Ohio |
| Frank Henry Fagan | Ohio |
| Joseph Wilbert George | Ohio |
| William Oscar Haldy | Ohio |
| Charles Emery Hurd | Ohio |
| John William Lewis Thomas | Ohio |
| John Sherman Van Meter | Ohio |

### Toronto Royal College of Dental Surgeons.

During the session of 1895–96 of the Royal College of Dental Surgeons of Toronto, Canada, there were one hundred and sixty-one students in attendance, viz.: Seniors, thirty-two; juniors, forty-seven, and freshmen, eighty-two.

The following named persons passed the final examination and received certificates of license to practice dentistry in Ontario, with the title of D. D. S.:

All of the Province of Ontario, Dominion of Canada.

J E Johnston

J M Bell

W F Templar

H A Croll

Percy Smith

H McQueen

W C Trotter, B.A.

T E Ball

C E Pearson

F Britton

J A Simpson

L M Mabee

J J Brown

W F Adams

E D Washington

R M Armstrong

O H Hutchison

L G Campbell

W G Switzer

W Burnet

C P Sherman

R H Henderson

J F McMillan

F S Mercer

J L Leitch

W E Lundy

A T Sihler

G W Hoag

J G Somerville

